# Multicomponent odd-parity superconductivity in UAu_2_ at high pressure

**DOI:** 10.1073/pnas.2210235119

**Published:** 2022-12-14

**Authors:** Christopher D. O’Neill, Julian L. Schmehr, Andrew D. Huxley

**Affiliations:** ^a^School of Physics and Astronomy and Centre for Science at Extreme Conditions, The University of Edinburgh, Edinburgh EH9 3FD, United Kingdom

**Keywords:** superconductivity, quantum criticality, magnetism, topological protection

## Abstract

We report that high-quality single crystals of the hexagonal heavy fermion material uranium diauride (UAu_2_) become superconducting at pressures above 3.2 GPa, the pressure at which an unusual antiferromagnetic state is suppressed. The antiferromagnetic state hosts a marginal fermi liquid and the pressure evolution of the resistivity within this state is found to be very different from that approaching a standard quantum phase transition. The superconductivity that appears above this transition survives in high magnetic fields with a large critical field for all field directions. The critical field also has an unusual angle dependence suggesting that the superconductivity may have an order parameter with multiple components. An order parameter consistent with these observations is predicted to host half-quantum vortices (HQVs). Such vortices can be topologically entangled and have potential applications in quantum computing.

## Significance

We report that an unconventional superconducting state is formed at high pressure in UAu_2_. The high critical field required to suppress superconductivity and its unusual anisotropy suggest that the superconductivity has multiple components. A detailed analysis suggests that it could potentially host mobile half-quantum vortices. Mobile half-quantum vortices analogous to those seen in confined superfluid ^3^He have never been observed in a superconductor to date but could potentially be manipulated to make topologically protected quantum computations unaffected by decoherence.

If the magnetic field penetrates into the bulk of any superconductor, it must do so in quantized bundles of flux. The quantization occurs because the superconducting order parameter has to be a single-valued function of position. It therefore has to wind through an exact number of turns around vortex cores where superconductivity is locally suppressed. Most superconductors have scalar order parameters having a phase that winds through a single turn giving a single quantum of flux. Half-quantum vortices (HQVs) could form in superconductors with order parameters that have two components. The two components together define a two dimensional (2D) vector-like order parameter $\vec{\eta }$. In this case, the winding could in principle be split equally between a winding of the overall phase and a rotation between the components of $\vec{\eta }$. The magnetic flux, which depends on the overall phase alone, is then halved. The crystalline symmetry for hexagonal (and also cubic and tetragonal) materials permits superconducting states with two components, but few such states are known. Further conditions, discussed later, also have to be satisfied to host HQVs.

HQVs would be highly interesting since they would obey non-Abelian statistics (1). Moving them around each other would generate quantum entanglement that could be exploited to make a robust quantum computer (2). Stable HQVs have been observed in geometrically confined superfluid helium-3 (3) and in polariton condensates (4). The observation of mobile HQVs in superconductors however remains elusive. Localized half-flux quanta have been detected in unconventional superconductors at tricrystal grain boundaries in the cuprates (5) and arguably in mesoscopic rings of Sr_2_RuO_4_ (6) and polycrystalline *β*-Bi_2_Pd (7). However, the noninteger flux in these cases is a consequence of the samples’ engineered geometry. Although we do not find direct evidence for bulk HQVs in UAu_2_, we find that UAu_2_ may be a favorable material in which to search for such exotic phenomena.

**Fig. 1.**
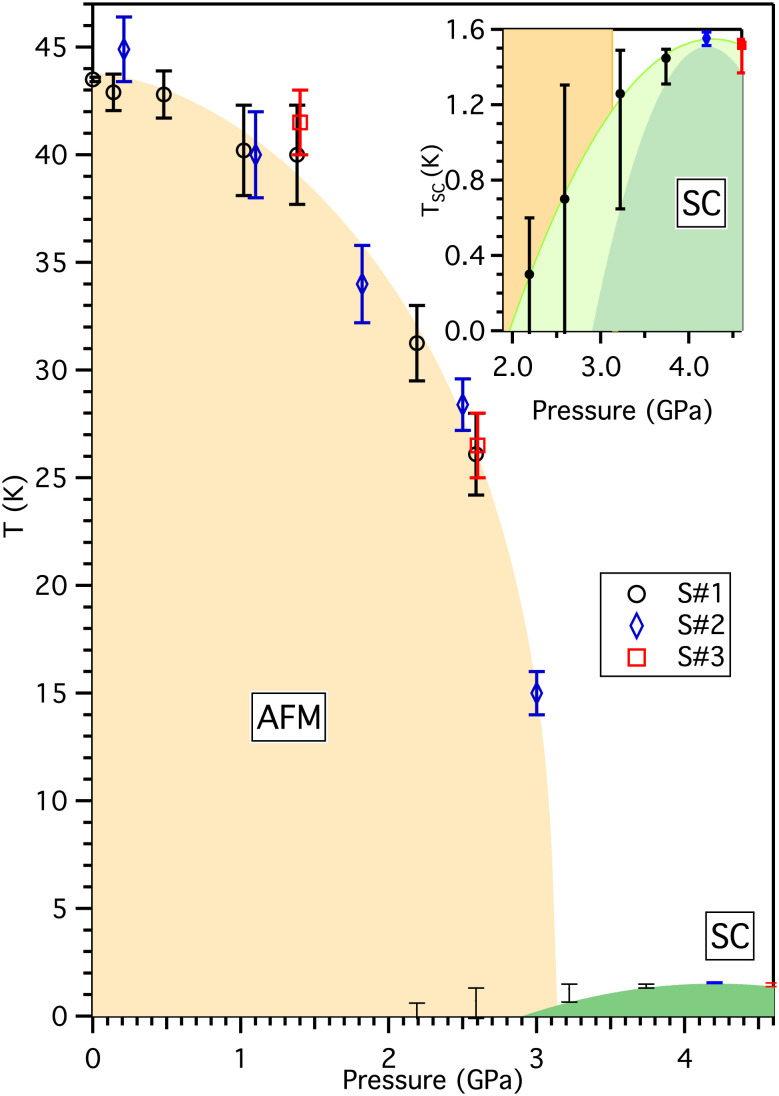
The temperature–pressure phase diagram for UAu_2_, determined from resistivity measurements. Three different samples denoted *S*#1, *S*#2, and *S*#3 were studied (black circles, blue diamonds, and red squares, respectively). The points above 10 K denote measurements of *T*_*N*_ and the points below 2 K denote superconductivity. For superconductivity, the top of the “error” bar is the point at which the resistivity first decreases from the normal state trend and the bottom marks the point at which the resistance is indistinguishable from zero. The inset shows the superconducting transition temperatures versus pressure in more detail. The points indicate where the resistance is 50% of the normal state value at the onset temperature. The resistivity curves are shown in Fig. 2. The resistivity does not drop to zero at 2.19 GPa and 2.59 GPa.

**Fig. 2.**
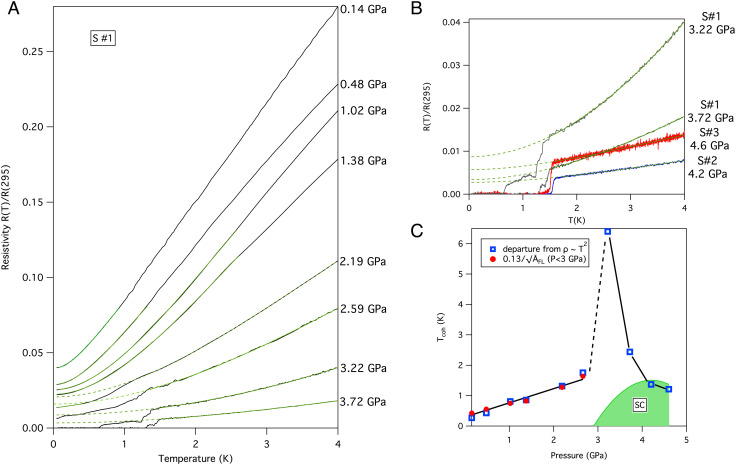
(*A*) The resistivity *ρ* of sample *S*#1 between 0.03 and 4 K, normalized to the value at 295 K, for different pressures. The solid green lines are fits described in the text, and the dashed green lines are extrapolations of these fits to low temperature that reveal partial or complete superconducting transitions. (*B*) Shows the data at two higher pressures measured on different samples *S*#2 and *S*#3. The resistivity at the two highest pressures measured for sample *S*#1 are replotted to aid comparison. In panel (*C*), the open circles show the estimated coherence temperature *T*_coh_ determined from the temperature at which the normal state resistivity deviates from a *T*^2^ dependence (see main text). The solid discs show *T*_coh_ deduced from $T_{\;\text{coh}}\sqrt{{\overset{¯}{A}}_{\mathrm{FL}}}=0.13$ with ${\overset{¯}{A}}_{\;\text{FL}}$ the low temperature *T*^2^ coefficient normalized to the room temperature resistivity. This gives consistent values in the AFM state below *P*_*C*_ ∼ 3.2 GPa but not above this pressure.

UAu_2_ has an AlB_2_-type hexagonal crystal structure. At room pressure, it orders antiferromagnetically (AFM) at *T*_N_ = 43 K, with moments directed along the *c*-axis (8, 9). We measured the electrical resistivity *ρ* of three different UAu_2_ single-crystal samples, denoted *S*#1, *S*#2, and *S*#3, under applied hydrostatic pressure. The pressure–temperature phase diagram deduced is shown in Fig. 1. *T*_N_ was determined from a kink in *ρ* (*SI Appendix*, Fig. S1). While present at 3 GPa, this feature is not identified at 3.22 GPa and above, locating the critical pressure *P*_*C*_ to suppress the AFM order just below 3.2 GPa. Full superconducting transitions are only seen at and above 3.22 GPa. At 3.22 and 3.72 GPa, the superconducting transition has two clear steps but has a sharper single step at higher pressure (Fig. 2*B*). There is a low temperature drop in the resistivity that suggest the presence of traces of superconductivity for 2 GPa <  *P* <  *P*_*C*_ (Fig. 2*A*). If the magnetic transition becomes first order at *P*_*C*_, this may give rise to some inhomogeneity accounting for the double-step transitions for *P* >  *P*_*C*_ and traces of superconductivity for *P* <  *P*_*C*_. This aspect of the phase diagram is similar to that for CeRhIn_5_ (10).

**Fig. 3.**
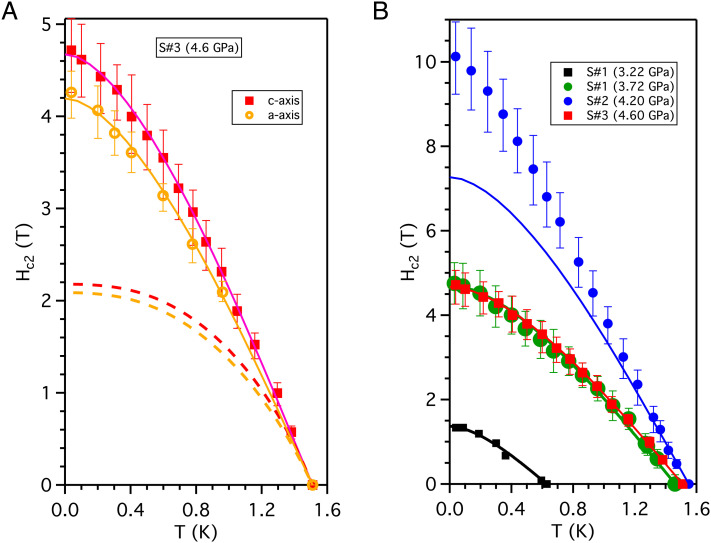
(*A*) *H*_*c*2_ against temperature at pressure 4.60 GPa (sample *S*#3) for field along the two principle axes. The critical fields are taken to be at the steepest point of the resistive transition and the error bars indicate the width of the transition (see *SI Appendix* for further details). The thick dashed lines show the BCS prediction for *H*_*c*2_ matching the slope *dH*_*c*2_/*dT* at *T*_*c*_. The solid lines are BCS curves without paramagnetic limiting and give a good description of the data. (*B*) *H*_*c*2_ for **H** ∥ **c**-axis at different pressures. The resistive transitions at 3.22 GPa are very broad, and for this pressure, the points at which the resistivity becomes zero are plotted. The solid lines show BCS fits ignoring paramagnetic limiting. A contact detached from *S*#2 (4.20 GPa) after the measurements shown and no further measurements were possible for other field directions for this sample. Unlike the other curves, this *H*_*c*2_ curve is not well described by the weak coupling BCS dependence in the absence of paramagnetic limiting (solid lines) but instead shows a weak upward curvature relative to this, indicative of strong coupling at this pressure.

Before discussing the superconductivity, we briefly discuss the evolution of the normal state properties with pressure, summarized in Fig. 2*A* for sample *S*#1. The measurements were made with current perpendicular to the *c*-axis. The room-temperature resistance changes by less than ∼1% with pressure. The absolute value of the low-temperature resistivity clearly decreases with pressure over the pressure range shown up to at least 3.72 GPa. For pressures below 2 GPa, where there is no trace of superconductivity, it is possible to identify a quadratic temperature dependence *ρ* = *ρ*_0_ + *A*_FL_*T*^2^ characteristic of a strongly correlated Fermi liquid (FL) at the lowest temperatures. At ambient pressure, the FL dependence was observed to cross-over at around *T*_coh_ ∼ 0.3 K to a non-Fermi liquid (NFL) behavior *ρ* = *ρ*_0_ + *A*_NFL_*T*^*n*^ with *n* ∼ 1.35 (valid up to at least 1.2 K) (11). A cross-over where the resistivity falls below *ρ*_0_ + *A*_FL_*T*^2^ is generally expected (12, 13) if *T*_coh_ is low as is the case here (for high *T*_coh_ scattering from phonons can obscure the fall). We find that this departure from a quadratic dependence under pressure is well-modeled phenomenologically for our measurements by *ρ* = *ρ*_0_ + *A*_FL_*T*^2^*e*^−*T*/*T*_0_^ up to *T* ∼ 0.7 *T*_0_ and take *T*_coh_ = *T*_0_/5. For *P* >  2 GPa, except at 3.22 GPa, the same dependence continues to describe the normal state, avoiding contamination from possible traces of superconductivity at low temperature. At 3.22 GPa, *T*_coh_ is relatively large; *T*_coh_ is defined in a consistent way as the temperature at which *ρ*(*T*)−*ρ*_0_ deviates by 18% (i.e., 1 − *e*^−0.2^) from the low-temperature form *A*_FL_*T*^2^. We find that both *ρ*_0_ and *A*_FL_ decrease with pressure in the AFM state. The product $A_{\;\text{FL}}^{1/2}T_{\;\text{coh}}$ is found to be approximately constant for *P* <  *P*_*C*_. The increase of *T*_coh_ and drop of *ρ*_0_ and *A*_FL_ with pressure approaching *P*_*C*_ (Fig. 2*C*) are opposite from the behaviors approaching most known quantum critical points (QCPs) (14). This confirms that the strong electronic correlations in the AFM state are not primarily driven by magnetic fluctuations linked to *T*_*N*_ but are instead intrinsic to the incommensurate state itself, as discussed in ref. 11. Just above *P*_*C*_ at 3.22 GPa, it is remarkable that the temperature dependence of the resistivity in the normal state remains quadratic to higher temperatures than at adjacent pressures, whereas for most QCPs, deviations from a fermi-liquid quadratic form would be clearest close to the critical pressure. The *T*^2^ resistivity at and just above *P*_*C*_ in UAu_2_ may therefore have a nonconventional origin, such as scattering from two soft boson modes (15–17). At pressures *P* >  *P*_*C*_, estimates of *A*_FL_ and *T*_coh_ are no longer consistent with the same constant value of the product $A_{\;\text{FL}}^{1/2}T_{\;\text{coh}}$. Since the resistivity is measured for different samples in Fig. 3*B*, differences in *ρ*_0_ at these higher pressures may reflect differences in sample quality.

We now discuss superconductivity. The transition temperature for unconventional superconductivity is reduced from the value in the pure limit by scattering from imperfections. The initial decrease is proportional to *ξ*/ℓ with ℓ the electron mean free path and *ξ* the coherence length, with *T*_*c*_ suppressed entirely for ℓ <  *ξ*. ℓ can be estimated from the normal state resistivity and *ξ* from the slope of the critical field at *T*_*c*_ (*SI Appendix* for details). These estimates show that the superconductivity is comfortably in the clean limit ℓ ≫ *ξ*. There is then no need to consider any weakening of the paramagnetic limit for the critical field due to strong impurity spin-orbit scattering, observed in the Chevrel phases (18).

The temperature dependence of the upper critical field *H*_*c*2_ for **H** ∥ *c*-axis and **H** ∥ *a*-axis at 4.6 GPa is shown in Fig. 3a (the supporting resistivity curves are given in the *SI Appendix*). For reduced dimensional materials, there may be special considerations such as those applied for quasi-one-dimensional Li_0.9_Mo_6_O_17_ (19) or for the 2D transition-metal dichalcogenides (20). However, these are not relevant to UAu_2_, which is a clean three-dimensional (3D) metal. Theoretically, *H*_*c*2_(*T*) is determined by both the orbital-limiting field *H*_orb_ and the Pauli paramagnetic limiting field *H*_P_. *H*_orb_ always dominates near *T*_c_ but below *T*_c_, *H*_*c*2_ can be more strongly limited if *H*_P_ is similar to or smaller than *H*_orb_. If paramagnetic limiting occurs, the shape of the *H*_*c*2_ curve is substantially modified. A para-magnetically limited *H*_*c*2_ curve is significantly more rounded at intermediate temperatures and flatter at lower temperatures than in the absence of paramagnetic limitation. Paramagnetic limiting is expected if i) *H*_orb_ is sufficiently large for the limit to be reached (i.e., *H*_orb_ ≳ *H*_P_) and ii) the Cooper pairs are formed from opposite spin states, projected along the field direction. For even parity singlet superconductivity, if the moment quantization direction is locked to a crystal axis, this second condition may result in paramagnetic limiting only for the field along this axis. Some forms of odd parity superconductivity are however able to avoid the paramagnetic limit for all field directions. We first discuss whether condition (i) is met for UAu_2_.

To make contact with experiment, we estimate *H*_orb_/*H*_P_ from the clean limit BCS formula *H*_orb_/*H*_P_ = 0.39 |*dH*_c2_/*dT*|_*T*_*c*__ (*SI Appendix*) with fields and temperatures in tesla and kelvin, respectively. The measured |*dH*_c2_/*dT*|_*T*_*c*__ for UAu_2_ gives *H*_orb_/*H*_P_ >  1 for all the pressures studied, so condition (i) is easily satisfied. The margin above 1 is sufficient for this conclusion to remain robust for modest coupling (strong coupling effects on the paramagnetic limit are discussed in the *SI Appendix*). We now look to see if there is evidence for paramagnetic limiting in our data to test condition (ii).

*H*_*c*2_(*T*) for a conventional BCS singlet state is completely determined from *T*_*c*_, and the slope of the critical field at *T*_*c*_. The calculated dependence is shown with dashed lines in Fig. 3*A*. The measured low temperature *H*_*c*2_(*T*) greatly exceeds the paramagnetically limited BCS dependence. This applies to all pressures, samples, and field directions studied. This is difficult to reconcile with a singlet (even parity) superconducting state, since for such a state, evidence of paramagnetic limiting would be expected along at least one direction. Except at 4.2 GPa, the curves are remarkably well accounted for by the weak-coupling BCS theory without paramagnetic limiting. Since strong coupling modifies the temperature dependence of *H*_*c*2_, this supports an interpretation in terms of weak coupling. Possible odd parity states are classified according to the irreducible representations (Irr Reps) of the crystal symmetry group (a full list is given in the *SI Appendix*). There are six possible Irr Reps, two of which describe superconductivity with two components (2D Irr Reps). These have order parameters denoted by a complex 2D parameter $\vec{\eta }$. The spin structure of the odd parity states is described by a complex 3D vector $\vec{d}$ at each position on the Fermi-surface. In the simplest case, the two components of $\vec{\eta }$ correspond to two different fixed directions of $\vec{d}$. When $\vec{d}$ is real, it points along the direction of zero spin of the Cooper pairs. This results in paramagnetic limiting when $H\|\vec{d}$. For a 2D Irr Rep, $\vec{d}$ may then be able to rotate (rotating $\vec{\eta }$) so that $\vec{d}$ remains perpendicular to **H**, avoiding paramagnetic limiting for all field directions. The observed lack of paramagnetic limiting for UAu_2_ is compatible with such a state.

**Fig. 4.**
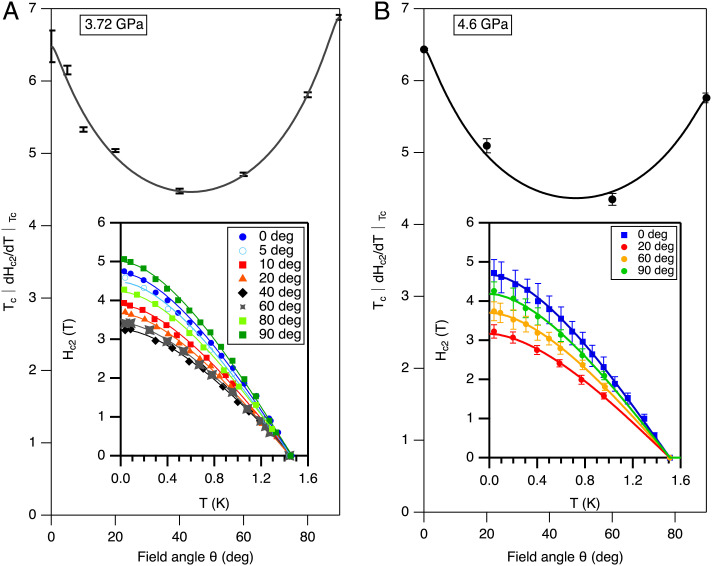
The main panels shows *T*_*c*_|*dH*_*c*2_/*dT*|_*T*_*c*__, the slope of the upper critical field at *T*_*c*_ multiplied by *T*_*c*_ as a function of the field angle from the c-axis measured at (*A*) 3.72 GPa and (*B*) 4.6 GPa. The critical field curves at different angles are shown in the insets. The lines through the measured critical field points are fits to the BCS form with no paramagnetic limiting. The lines in the main panel are guides to the eye (obtained from a phenomenological formula (21)).

To narrow down the choice of order parameter further, we measured the angle dependence of the critical field for field directions between the *c*-axis and basal plane at 3.72 and 4.60 GPa. We find that *H*_*c*2_(*T*) at all angles continues to be well described by the BCS form without paramagnetic limiting (Fig. 4). The angle dependence of *T*_*c*_|*dH*_*c*2_/*dT*|_*T*_*c*__ is shown in Fig. 4. There is a small change in the effective mass anisotropy |*dH*_*c*2 ∥ *c*_/*dT*|/|*dH*_*c*2 ∥ *a*_/*dT*| with pressure. However, the main feature is a strong minimum of *H*_*c*2_(*θ*) at intermediate angles, present at both pressures, extending up to *T*_*c*_. This is characterized by a dip in *T*_*c*_|*dH*_*c*2_/*dT*|_*T*_*c*__ at intermediate angles, which is highly unusual and the focus of our discussion.

The increase of the critical field approaching *θ* = 0° and *θ* = 90° invites a comparison with the cusp-like angle dependence of the critical field for surface superconductivity *H*_*c*3_ (21). We therefore examine first whether the critical field we measure is a measurement of *H*_*c*2_ or *H*_*c*3_. Surface superconductivity is still subject to paramagnetic limiting (22, 23), so the conclusion that the superconductivity has odd parity would be unchanged. Surface superconductivity for odd-parity states is suppressed for diffuse scattering at surfaces unless the surfaces are atomically flat. Our samples are discs with their large faces spark-cut and therefore rough. *S*#1 is a disc normal to **c**, and *S*#3 is a disc normal to **a**. The electrical contacts are on the rough faces. The transitions we see cannot be confined to the cleaved edge of the discs since the resistivity drops to zero, not a finite value. It thus appears the critical fields measured must be the bulk critical field *H*_*c*2_.

Anisotropy of *H*_*c*2_ can in general arise from anisotropy of the Fermi-surface and of the superconducting gap (24). As *T* → *T*_*c*_, this source of anisotropy reduces to an effective mass form that has a monotonic dependence on *θ* (*SI Appendix* for details). The above sources of anisotropy also give rise to nonmonotonic contributions to *H*_*c*2_(*θ*) (known as nonlocal corrections), but these disappear linearly with temperature or more strongly as *T* → *T*_*c*_ (*SI Appendix*) (25). Our data does not follow this predicted behavior; we find a nonmonotonic dependence (with a clear minimum of *H*_*c*2_(*θ*)/*H*_*c*2_(0) at around *θ* ∼ 45°) that is almost independent of temperature, persisting up to at least *T*/*T*_*c*_ ∼ 0.9. A nonmonotonic anisotropy for *H*_*c*2_(*θ*) that persists to *T*/*T*_*c*_ = 1 is predicted for superconductors with a two-component order parameter. The two components may arise for a 2D Irr Rep or possibly due to two very weakly coupled order parameters with accidentally degenerate *T*_*c*_. We next examine these two possible sources of nonmonotonic anisotropy in turn.

For a 2D Irr Rep, assuming strong spin-orbit coupling, the spin pairing vector $\vec{d}\left(k\right)=\eta_{x}{\vec{d}}_{1}\left(k\right)+\eta_{y}{\vec{d}}_{2}\left(k\right)$, where ${\vec{d}}_{1}\left(k\right)$ and ${\vec{d}}_{2}\left(k\right)$ are orthogonal states with degenerate *T*_*c*_. Zhitomirsky (26) studied the angle dependence of *H*_*c*2_ for a field inclined between **H** ∥ **c** and **H** ⊥ **c** and established that a nonmonotonic *H*_*c*2_(*θ*) is possible in the limit *T* → *T*_*c*_. For **H** ∥ **c**-axis, the order parameter is built from the states (*η*_*x*_, *η*_*y*_)∝(1, ±*i*). For **H** ⊥ **c**, the components *η*_*x*_, *η*_*y*_ do not mix, resulting in a state where one component is zero (26, 27). A nonmonotonic *H*_*c*2_(*θ*) arises when anisotropy due to the order parameter transforming between these two limits has an opposite angular dependence from the effective mass anisotropy. We explored the anisotropy for different parameters imposing that experimentally *H*_*c*2∥_ = *H*_*c*2⊥_. A dip in *H*_*c*2_(*θ*) at intermediate angles occurs over a wide range of parameters with a maximum dip (within the range the parameters give stable solutions) of *δH*_*c*2_(*θ*)_min_/*H*_*c*2_(0)∼ − 12% (details are given in the *SI Appendix*). A larger dip would require higher-order gradient terms to be included in the analysis to ensure stability. The experimental dip is ∼−30%.

We now consider two order parameters belonging to the same Irr Rep. The mass anisotropy for each of the uncoupled order parameters is determined by a combination of a Fermi surface and gap anisotropy (1/*m*)_*ij*_ ∝ ⟨(*v*_*f*_)_*i*_(*v*_*f*_)_*j*_*Δ*(*k*)^2^⟩ (*i*, *j* label the Cartesian axes, *Δ*(*k*) is the gap, **v**_*f*_ is the Fermi velocity and **k** is the momentum direction of each point on the Fermi-surface). A Josephson-type coupling *ϵ* mixes the order parameters (gradient couplings are also allowed but are rarely considered). This description has been widely applied to describe two-band superconductivity, including for MgB_2_ (28) and NbSe_2_ (29). We note that Askerzade and Tanatar (21) give a phenomenological analytic expression for *H*_*c*2_(*θ*) in a two-band model that includes a gradient coupling term. Even setting the gradient coupling parameter in their expression to zero, parameters can be chosen that give an excellent fit to our *H*_*c*2_(*θ*) data. Here, we consider the case with an accidental degeneracy for *T*_*c*_ in the absence of coupling. If *ϵ* = 0, the critical field is determined by the higher of two independent critical fields which can swap over as a function of field angle, resulting in a dip in *H*_*c*2_(*θ*)/*H*_*c*2_(0) at intermediate angles. For effective mass anisotropies of *m*_*zz*_/*m*_*xx*_ = *m*_*zz*_/*m*_*yy*_ = 3 and 1/3 for the two order parameters, a dip in *δH*_*c*2_(*θ*)/*H*_*c*2_(0)∼ − 30% at *θ* = 45° is obtained. For *ϵ* ≠ 0, *H*_*c*2_ can be found numerically (*SI Appendix* for details) (28). We find a nonzero *ϵ* suppresses the dip very close to *T*_*c*_ and results in a strong temperature dependence of the anisotropy. However, if *ϵ* is sufficiently small, the suppression of the dip in *H*_*c*2_(*θ*) may occur too close to *T*_*c*_ to be observable experimentally. Polar states like *Δ* ∝ *k*_*z*_ and composed from *Δ* ∝ *k*_*x*_ and *Δ* ∝ *k*_*y*_ are particularly interesting since these have effective mass anisotropies of the above values for isotropic Fermi-surfaces. Amongst the Irr Reps for UAu_2_, the 2D Irr Rep *E*_1*u*_ permits purely polar order parameters of these forms. Although two 2D *E*_1*u*_ order parameters accessing solutions with $\vec{d}$ parallel and perpendicular to the c-axis could provide an explanation of our data, an accidental degeneracy between $\vec{d}\|c$ and $\vec{d}⊥c$ states appears rather contrived. We return to this point later.

We next examine the pressure evolution of the superconductivity. At 4.2 GPa, |*dH*_*c*2_/*dT*|_*T*_*c*__ is significantly higher than at adjacent pressures 3.72 GPa and 4.6 GPa (Fig. 3*B*). The increase exceeds that predicted from the change of *T*_*c*_ (|*dH*_*c*2_/*dT*|_*T*_*c*__ ∝ *T*_*c*_) for constant coupling and effective mass (valid for different impurity contents). There is therefore a strong peak in the electronic effective mass and/or coupling strength near 4.2 GPa. The *H*_*c*2_ curve at 4.20 GPa while also showing no sign of paramagnetic limiting has a different allure from the nonparamagnetically limited BCS form at other pressures, with a weak upward curvature, suggestive of stronger coupling at this pressure. The increase in *T*_*c*_ with pressure from 3.22 GPa to 4.2 GPa is larger than can easily be accounted for by differences in pair breaking due to scattering from imperfections, proportional to *ξ*/ℓ (*SI Appendix*). However, the small fall in *T*_*c*_ between 4.2 GPa and 4.6 GPa is less clear cut and may be due to the increased residual resistivity of the higher pressure sample. An increase in *T*_*c*_ and stronger coupling at a pressure which is above and distinct from *P*_*C*_ is reminiscent of the pressure dependence theoretically expected above the critical pressure for suppressing isotropic ferromagnetism (FM) (30) (different from uniaxial FM encountered in the ferromagnetic superconductors like UGe_2_). For an isotropic ferromagnetic QCP, superconductivity is suppressed exactly at the QCP due to the presence of soft transverse modes, resulting in a maximum *T*_*c*_ and coupling strength at a higher pressure. Thus, for UAu_2_ it also appears that critical fluctuations at *P*_*C*_ are both pair breaking and pair forming.

We now briefly compare our findings on paramagnetic limiting and the unusual angular dependence of *H*_*c*2_ with those for other recently discovered uranium-based superconductors. Large critical fields exceeding the BCS Pauli paramagnetic limit have been found in a number of materials where odd parity pairing has been additionally confirmed by NMR. These include the ferromagnetic superconductors UGe_2_ (31), URhGe (32), UCoGe (33), and most recently in a related material UTe_2_ (34). Although the low-temperature critical fields for the ferromagnetic superconductors perpendicular to their easy-axis are large compared with the paramagnetically limited BCS critical field, this is not always the case for field parallel to their ordered moments (35). All of these materials also have very unusual field reentrant *H*_*c*2_ curves for at least one field direction, attributed to field tuning of the pairing interaction. This further complicates the identification of paramagnetic limiting. The reentrance gives a sharply peaked angular dependent *H*_*c*2_, but reasonably close to *T*_*c*_, the angular dependence in all cases is experimentally described by a simple effective mass anisotropy. This is expected since the crystal symmetry of these materials is orthorhombic, so the only states available to them are one-dimensional (1D) Irr Reps.

Returning to UAu_2_, we have established that the observed dip in *H*_*c*2_ at intermediate field angles can potentially be described starting from a 2D Irr Rep. This begs further comparison with UPt_3_ which is hexagonal and has an order parameter widely accepted to belong to the 2D *E*_2*u*_ Irr Rep. The magnetic susceptibility of UPt_3_ has *χ*_*a*_ >  *χ*_*c*_ which locks $\vec{d}$ to the **c**-axis. There is then no paramagnetic limiting for **H** ∥ **a**, but there is for **H** ∥ **c** (36). For UAu_2_ at low pressure, we have *χ*_*c*_ >  *χ*_*a*_, which would suggest $\vec{d}⊥c$. If $\vec{d}$ can rotate within the basal plane (for example, in the *E*_1*u*_ Irr Rep), paramagnetic limiting is avoided for all field directions.

The lowering of hexagonal symmetry by antiferromagentic order in UPt_3_ breaks the degeneracy of the 2D Irr Rep and results in two transitions in zero field. The order parameter then has a single component close to the higher transition temperature *T*_*c*+_ which explains why UPt_3_ has a simple monotonic *H*_*c*2_(*θ*) close to *T*_*c*+_ (37), unlike UAu_2_.

The uniform free-energy with no symmetry breaking, expanded up to quartic terms of the order parameter (valid for both possible 2D Irr Reps *E*_1*u*_ and *E*_2*u*_), is


$$F_{\;\text{uniform}}=\alpha \left(T\right){\vec{\eta }}^{*}.\vec{\eta }+\beta_{1}{\left({\vec{\eta }}^{*}.\vec{\eta }\right)}^{2}+\beta_{2}{\left|\vec{\eta }.\vec{\eta }\right|}^{2}.$$

For weak coupling *β*_2_/*β*_1_ = 0.5 if $\vec{d}\|c$, whereas *β*_2_/*β*_1_ = −0.5 if $\vec{d}⊥c$ (*SI Appendix*). This leads to significant differences in the predicted behavior. For *β*_2_/*β*_1_ >  0 (UPt_3_), the free energy in zero field is minimized for $\vec{\eta }\propto \left(1,\pm i\right)$, whereas for *β*_2_/*β*_1_ <  0 (UAu_2_), it is minimized when $\vec{\eta }$ is a real vector-like quantity that is free to rotate in the basal plane just below *T*_*c*_ (at lower temperatures, terms in *F*_uniform_ of higher power in *η*, known as nonlocal corrections, induce a sixfold anisotropy).

Although both materials have the same point group, UPt_3_’s crystal structure has two inequivalent U sites per unit cell compared with one in UAu_2_. For UPt_3_, inequivalent sites are considered a necessary ingredient to form odd-parity superconductivity with AF fluctuations (38). An odd parity state in UAu_2_ would require FM fluctuations. The AFM state for *P* <  *P*_*C*_ in UAu_2_ however has a long incommensurate modulation period (at room pressure) which locally (on the length scale of the coherence length) resembles ferromagnetism. Microscopic theories (38, 39) tend to favor an *E*_1*u*_ symmetry over *E*_2*u*_ since short-range interactions favor lower orbital spherical harmonics (the microscopic mechanism leading to an *E*_2*u*_ state in UPt_3_ remains unclear).

To summarize, in UAu_2_, we found an increase of *T*_coh_ in the AFM state with pressure and surprisingly a *T*^2^ resistivity over a wide temperature range at the critical pressure *P*_*C*_ at which AFM is suppressed. This behavior is highly unusual, and the opposite of the standard behavior crossing most known quantum phase transitions. It may indicate the presence of multiple soft boson modes. Superconductivity occurs with a higher *T*_*c*_ and stronger coupling at a pressure that is distinctly above *P*_*C*_. Such a pressure dependence is similar to that theoretically predicted above a QCP for suppression of isotropic ferromagnetism (30), where soft modes at *P*_*C*_ are both pair breaking and pair forming. The long period of modulation along the c-axis in the AFM state (at zero pressure) may indeed locally appear FM-like on the scale of the superconducting coherence length. The high critical field for superconductivity along all axes, which exceeds the weak coupling paramagnetic limit, indicates that the superconductivity most likely has an odd-parity order parameter, with $\vec{d}$ able to rotate away from the field direction. An unusual minimum of the critical field with an inclination of the field from the c-axis was found, which can be explained if the order parameter has two components. While the large magnitude of the observed dip exceeds the simplest predictions for a 2D Irr Rep, based on Ginzburg–Landau theory, this theory is able to account for a sizeable dip. We also explored a related model with an accidental degeneracy between two uncoupled *E*_1*u*_ states, one with $\vec{d}⊥c$ and a second with $\vec{d}\|c$. It is possible that such an “accidental coincidence” and the destruction of the AFM are related, for example, if both are driven by a common mechanism such as a reduction of magnetic anisotropy with pressure. Indirect evidence for a reduction in magnetic anisotropy with pressure comes from the transition to ferrimagnetism seen at room pressure at around 6 T for **H** ∥ **c**. The transition field increases with pressure (*SI Appendix*, Fig. S5) indicating that aligning all the moments to the zero pressure easy c-axis becomes more difficult with increasing pressure, requiring higher fields.

Our measurements of *H*_*c*2_ point toward an order parameter belonging to a 2D Irr Rep with $\vec{d}⊥c$. Such a state is an ideal candidate for hosting HQVs. For weak coupling, it would have Ginzburg–Landau parameters *β*_2_/*β*_1_ = −0.5. For *β*_2_/*β*_1_ ⪆ −0.5 (achieved for example if $\vec{d}$ is titled slightly toward the *c*-axis), single quantum vortices are theoretically predicted to disassociate into HQVs (40) that separate on the length scale of the penetration length (of order μm). Our work thus identifies UAu_2_ as a prime candidate material in which to search for half-quantum vortex formation.

The discovery that UAu_2_ becomes superconducting at high pressure provides an important addition to a limited number of recently discovered exotic superconductors. Classifying and understanding how such superconducting states form is an exciting direction for future research.

## Materials and Methods

Single crystals of UAu_2_ were grown following the method described in ref. 11. Samples *S*#2 and *S*#3 are different pieces of the same parent crystal. The cut samples had approximate dimensions 200 μm × 150 μm × 80 μm with gold wires for resistivity measurements spot welded along their lengths in a standard four-point geometry. The pressure cell was a hardened beryllium copper Merrill–Bassett cell with opposing 800-μm culet diamond anvils and a 200-μm thick stainless steel gasket, preindented to 110 μm, electrically insulated with an Al_2_O_3_ powder and Stycast epoxy 1266 mixture. The liquid pressure medium was Daphne oil 7373. The pressure was determined at room temperature from the fluorescence of a small ruby chip located next to the sample. No measurable changes in pressure were observed upon thermal cycling. Electrical resistivity was measured with a constant amplitude 100 μA (rms) a.c. current at 37 Hz and synchronous voltage detection. For temperatures 2 to 295 K, the measurements were made in a continuous flow cryostat, and the voltage was amplified with a low-noise transformer (SRS SR554, Gain 100). For temperatures in the range 0.03 to 4 K, the measurements were made in a dilution refrigerator with a low-temperature transformer (CMR Ltd, Gain 30, located on a regulated 5K plate). The data have been deposited in the Edinburgh DataShare digital repository (https://doi.org/10.7488/ds/3784).

## Supplementary Material

Appendix 01 (PDF)

## Data Availability

All study data are included in the article and/or *SI Appendix*.
