# Supplementary material for: Multicomponent odd-parity superconductivity in UAu2 at high pressure
Source: Proc Natl Acad Sci U S A. 2022 Dec 14;119(51):e2210235119. doi: 10.1073/pnas.2210235119 (PMC9907152; doi:10.1073/pnas.2210235119)
Supplement: Supplementary file 1 — Appendix 01 (PDF) [file pnas.2210235119.sapp.pdf]

Multicomponent odd-parity superconductivity in UAu_2 at high pressure: supplementary material

Christopher D. O'Neill[1], Julian L. Schmehr[1], and Andrew D. Huxley[1]

[1]School of Physics and Astronomy and Centre for Science at Extreme Conditions, The University of Edinburgh, Mayfield Road, Edinburgh, EH9 3FD, UK.

Determination of T_N

T_N shown in FIG 1 was determined from the measured resistivity, shown in FIG S1 for sample $S\#1$ (and similar measurements on the other samples).

For pressure $P > P_C$ there is a peak in $d\rho/dT$ at around $T^* \sim 20$ K (FIG S1). It is remarkable that a feature at the same temperature $T^* \sim 15 - 20$ K is also identified in the the zero pressure study[1] linked to a change in the lattice expansion. This may be hidden by the upturn in the derivative of the resistivity below T_N at lower pressure.

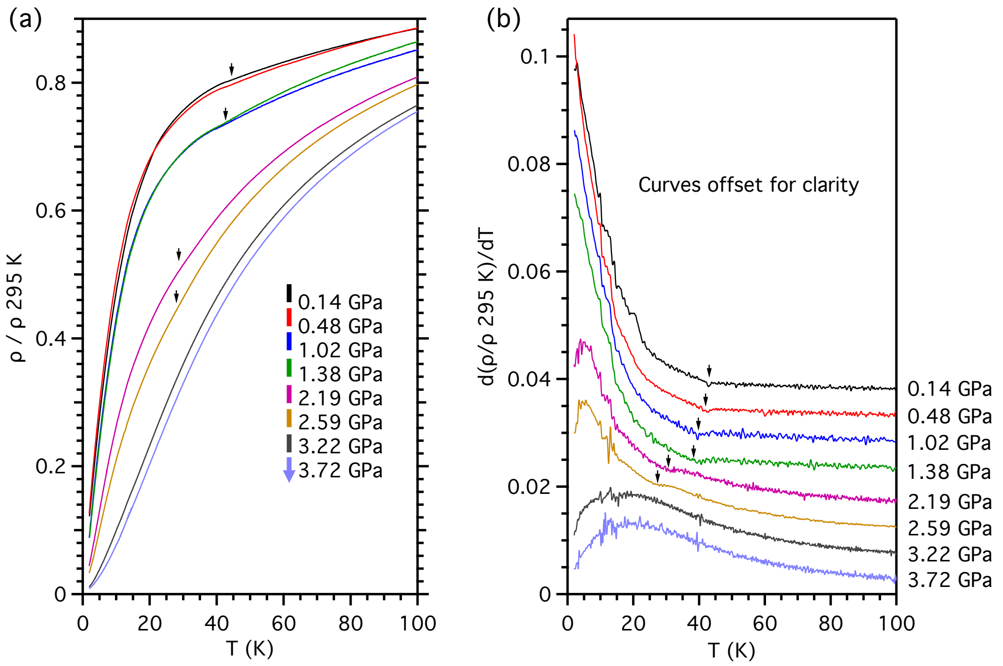

Figure S1: (a) The resistivity of sample $S\#1$ at a series of pressures, normalised to the value at 295 K. The value at 295 K remained constant within 1% for all pressures. (b) The derivative of the resistivity. The value of T_N was determined from a kink indicated by the black arrows. For pressures at and above 3.22 GPa, T_N is no longer detectable.

Critical field curves for sample $S\#1$ at 3.72 GPa and 3.22 GPa

FIG S2 shows the resistive superconducting transitions for sample $S\#1$ at 3.72 GPa in different fields and the field dependence of different features in the transition, for field along both principle axes. In

zero field there are two steps in the transition. For **H** ∥ **a** the two steps, which we refer to as a foot and main transition track each other. For **H** ∥ **c** with decreasing temperature the foot appears to merge with the main transition and then a broad shoulder emerges at lower temperature (the field dependence of both these features are shown with open symbols). The main step in the resistivity remains clearly defined and is shown by the solid symbols in the figures. It is clear from the data close to T_c that the solid symbols for both directions represent the same transition and provide a consistent identification for H_{c2}. A similar trend is seen in the angular dependence (shown at low temperature in FIG S3(a)-(g)). The temperature dependence of the form of the transition for $\theta \leq 20^o$ is similar to **H** ∥ **c**, while for $\theta \geq 60^o$ it is similar to **H** ∥ **a** (at 60° the main transition and foot seen at higher angle are very close together and not easily resolvable except close to T_c). At a field angle of 40° to **c** the foot is present close to T_c, but there is no shoulder at low temperature. At smaller angles the sharp transition at H_{c2} is easily identified and distinguished from the broad shoulder at higher field. The critical field for the main transition is plotted in FIGS 3(b) and 4(a) (main text). The transition fields are obtained as the point of maximum slope of the resistivity against field and the transition width shown as an error bar is the Gaussian width of the peak in the derivative. Similar results are obtained defining H_{c2} with other criteria, such as at the midpoint of the transition.

At 3.22 GPa measurements on sample $S\#1$ were only made for **H** ∥ **c**. The transition has two well separated distinct steps in zero field at this pressure, although in detail each of these steps could comprise further broad and sharp jumps (FIG 2). The low temperature onsets of the main steps are at 0.63 K and 1.23 K. These distinct features remain defined in field down to low temperature, giving corresponding critical fields 1.33 T and 4.40 T. The large width of the overall transition and limited data at this pressure restrict a more quantitative analysis. The critical field for zero resistance is plotted in FIG 3(b).

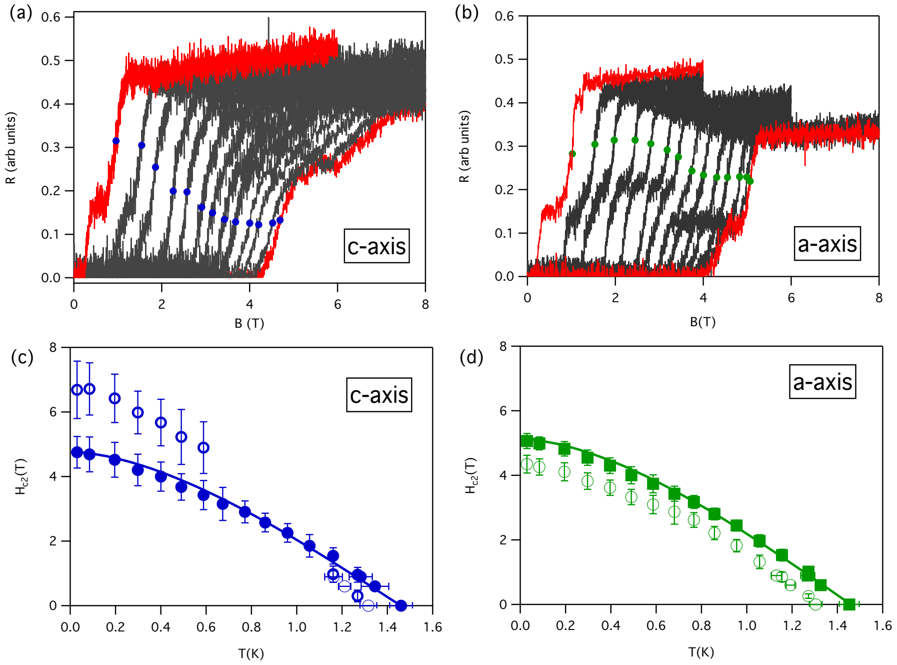

Figure S2: The resistance at different temperatures as a function of field for sample $S\#1$ at pressure 3.72 GPa with (a) **H** ∥ **c** (temperatures 85 mK and 1268 mK in red) and (b) $H \parallel a$ (29 mK and 1272 mK in red). Panels (c) and (d) show the deduced upper critical field at the inflection point of the sharp step in the resistance (solid symbols). For **H** ∥ **c** there is an additional step at the foot of the transition for higher temperatures and shoulder above the main transition for lower temperatures (the inflection points defining these features are shown by open symbols). For $H \parallel a$ only a foot is present. The solid lines in (c) and(d) are BCS curves without paramagnetic limiting. The H_{c2} data are very similar to those at 4.6 GPa shown in the main text (FIG 3a). At 4.6 GPa there is a single transition.

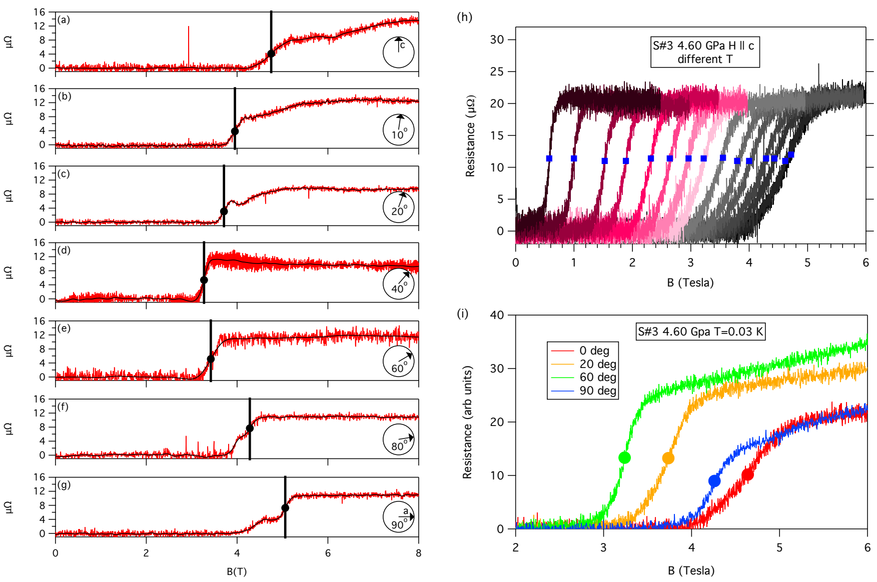

Figure S3: (a)-(g) Resistance curves of sample $S\#1$ at 3.72 GPa and 0.03 K for different applied field directions between the **a** and **c**-axes. H_{c2} is marked by black vertical lines. The dip in H_{c2} at intermediate angles is clear and is plotted in FIG 4a. (h) Resistance against field curves for sample $S\#3$ at 4.60 GPa for **H** $\parallel$ **c** at different temperatures. The values of H_{c2} are shown by squares and are plotted against temperature in FIG 3. (i) Resistance curves at 0.03 K for sample $S\#3$ at 4.60 GPa for different angles of the field from the c-axis. The position of the critical field is shown with solid circles. The dip in critical field at intermediate angles is clear. H_{c2} versus temperature curves at different angles are shown in FIG 4b.

Critical field curves for sample $S\#3$ at 4.60 GPa

Resistive transitions for sample $S\#3$ at 4.60 GPa with **H** $\parallel$ **c** are shown in FIG S3(h). There is a single transition for this sample at this pressure. Resistance curves for different field angles at 0.03 K are shown in FIG S3(i). The position of the transition is taken to be at the inflection of the curves, obtained by fitting the derivative of the resistivity to a Gaussian (error bars show the width σ of the Gaussian, and are therefore overestimates of the error in relative changes of the critical field). Other criteria for defining the transition field (e.g midpoint) give similar results within these error bars. The overall magnitude of the critical field is substantially reduced for the field inclined at 60° to **c** relative to its value at **H** $\parallel$ **c** and **H** $\parallel$ **a**. The temperature dependence of the critical field at the different field angles and the angle dependence of $T_c(dH_{c2}/dT)_{T_c}$ are plotted in FIG 4(b) (main text).

Estimation of the electron mean free path and its effect on T_c

We consider pressures $P > P_C$. The coherence length can be estimated from the BCS theory applied to the critical field slope for **H** $\parallel$ **c** at T_c. From $T_c(dH_{c2}/dT)_{T_c} = 1.43\ \phi_0/2\pi\xi^2$, $\xi = 85,\ 67(\pm 7)$ and 85Å at 3.72, 4.2 and 4.6 GPa (the values for **H** $\parallel$ **a** are similar, the error at 4.2 GPa is larger than at the other pressures since the full non-paramagnetically limited BCS dependence cannot be used to more accurately determine the critical field slope for this pressure).

The fermi-velocity is estimated from $v_f = 2\pi k_B T_c \xi/\hbar \sim 10200,\ 8520(\pm 800),\ 10600\ \text{ms}^{-1}$ at the same pressures. The apparent dip at 4.2 GPa is most probably a consequence of stronger coupling at this pressure. Stronger coupling increases the slope of the critical field relative to the BCS formula[2,3]. Thus the actual v_f at 4.2 GPa could be aligned with the values at the two adjacent pressures.

The electron-mean-free path ℓ is estimated from the free electron result $\ell = \frac{3\pi^2\hbar}{e^2(3\pi^2 n)^{2/3}\rho_0}$ with n the electron density and ρ_0 the residual resistivity. The residual resistivity values are 0.26, 0.21 and 0.43 $\mu\Omega$cm from FIG 2 and a room temperature resistivity[1] 76 $\mu\Omega$cm $\pm$ 10%. Taking n to be one electron per cell gives $\ell = 7600, 9400$ and 4600Å at 3.72, 4.2 and 4.6 GPa respectively. The superconductivity is thus comfortably in the clean limit $\ell \gg \xi$ at all these pressures.

The suppression of T_c from that for a perfect sample is[4] $\Delta T_c = -\frac{\pi}{8}\frac{\hbar}{k_B\tau}$ where the scattering time is

$\tau \sim \ell/v_f$. With the above estimates this gives $|\Delta T_c| \sim 41$, 27 and 69 mK at 3.72, 4.2 and 4.6 GPa. The experimentally observed increase of T_c with pressure from 3.72 GPa to 4.2 GPa exceeds the difference in $|\Delta T_c|$, but the small decrease in T_c from 4.2 GPa to 4.6 GPa might be due to a change in electron mean free path.

The paramagnetic limit

The zero temperature paramagnetic limit H_{P} is taken to be the Clogston-Chandrasekhar[5] value $H_{\mathrm{P}} = 1.85\ T_c$ (with units Tesla and Kelvin). A slightly higher value is derived for a clean weak-coupling conventional superconductor if a modulation of the superconductivity along the field direction is included in the analysis[6] (a lower value is obtained from the condition for a continuous transition without modulation[2]). An enhancement of the normal state spin susceptibility above the Pauli value would lower H_{P}. In the main text the ratio $\frac{H_{\mathrm{orb}}}{H_{\mathrm{P}}} = 0.392 \left|\frac{dH_{c2}}{dT}\right|_{T_c}$ follows from $H_{\mathrm{P}} = 1.85\ T_c$ and the standard clean limit BCS result $H_{\mathrm{orb}} = -0.727\ T_c \left(\frac{dH_{c2}}{dT}\right)_{T_c}$ in the absence of paramagnetic limiting.

The paramagnetic limit in the presence of strong coupling

Paramagnetic limiting of the critical field is present when the dimensionless Maki parameter[7] $\alpha = \frac{\sqrt{2}H_{\mathrm{orb}}}{H_{\mathrm{P}}} \gtrsim 1$. For weak coupling BCS theory $\alpha \sim \frac{gm^*}{m}\frac{\Delta}{E_{\mathrm{F}}}$ where m^*/m is the ratio of the electronic mass to the free electron mass. The electronic mass includes both the effect of band structure and interactions. $\Delta \sim k_B T_c$ is the average superconducting energy gap, E_{F} is the Fermi-energy and g is the electron g-factor. $g = 2$ for free electrons. A smaller value for g is unlikely for UAu_2 since at ambient pressure the Curie-Weiss moment is close to that for isolated uranium ions $\sim 3.5\mu_B$, so that any corrections to the effective g are likely to increase it from 2. For conventional metals with $\frac{m^*}{m} \sim 1$, fulfilling the condition $\alpha \gtrsim 1$ requires strong coupling $(\Delta/E_{\mathrm{F}}) \gtrsim 1$. For heavy fermion metals like UAu_2, strong coupling is not necessary to get $\alpha \gtrsim 1$, since $\frac{m^*}{m} \gg 1$. Firstly, the band mass is expected to be much larger than the bare electron mass due to narrow f-electron bands. Secondly, $\frac{m^*}{m}$ may be enhanced further by critical fluctuations. The observation that T_c is maximum away from P_C in UAu_2 suggests that fluctuations may be both pair breaking and pair forming[8] and so these may enhance $\frac{m^*}{m}$ without necessarily also enhancing the superconducting coupling strength.

Moderate coupling increases H_{P} relative to H_{orb} reducing α. The fractional increase in H_P relative to its weak coupling value is of the order $(1+\lambda)$ with λ a dimensionless coupling constant[2]. UBe_{13} provides an example of a heavy fermion material where very strong coupling was thought to increase H_{P} above $1.85\ T_c$ avoiding the paramagnetic limit for a singlet state with very high critical fields [9,10], although recent measurements under pressure suggest the superconductivity is an odd-parity state [11]. In UBe_{13} the possibility of very strong coupling is evident from the strong field dependence of the normal state properties and because $T_c \gg T_{\mathrm{coh}}$. Theoretically, very strong coupling results in an upward curvature in the temperature dependence of H_{c2}[3], as measured for UBe_{13}. For UAu_2 although we have $T_c \gtrsim T_{\mathrm{coh}}$, any upward curvature of $H_{c2}(T)$ is rather weak and confined to the measurements at 4.2 GPa. Another interesting material where strong coupling has been suggested to apply is $CeCoIn_5$[12]. At zero pressure it is argued that the value for H_{P} is increased above $1.85\ T_c$ due to strong coupling, but the critical field is high enough that paramagnetic limiting still occurs. The resulting H_{c2} curves are quite different from the non-paramagnetically limited BCS form. These examples show that although strong coupling can raise the paramagnetic limit above the Clogston-Chandrasekhar value, the resultant $H_{c2}(T)$ curves differ significantly from the weak coupling non-paramagnetically limited BCS form. The observation of a weak coupling non-paramagnetically limited BCS temperature dependence for UAu_2 at 3.72 GPa and 4.6 GPa with critical fields above the Clogston-Chandrasekhar limit cannot therefore be easily explained for singlet pairing with strong coupling.

Effective mass and non-local corrections to the anisotropy of H_{c2}.

The expression for $H_{c2}(\theta)$ with an anisotropic effective mass is

$$H_{c2}(\theta) = \frac{\phi_0(1 - T/T_c)}{2\pi\sqrt{\xi^4_{GL\perp}\cos^2(\theta) + \xi^2_{GL\perp}\xi^2_{GL\parallel}\sin^2(\theta)}}.$$

ϕ_0 is the flux quantum, $\xi_{GL\parallel}$ and $\xi_{GL\perp}$ are the Ginzburg-Landau coherence lengths parallel and perpendicular to $\mathbf{c}$, and θ is the angle of the field from $\mathbf{c}$[13] (the anisotropy of the coherence length and effective mass are related by $\xi_{GL\parallel}/\xi_{GL\perp} = \sqrt{m_\perp/m_\parallel}$). The expression for $H_{c2}(\theta)$ has a monotonic dependence on θ.

Corrections to the effective mass form can be non-monotonic in θ and arise from Fermi-surface and gap anisotropy. In the Ginzburg-Landau expansion these corrections come from higher order than quadratic derivatives in the free energy[14]. These corrections contribute corrections to the effective mass expression for H_{c2} that grow as $(1 - T/T_c)^2$ and higher powers of $(1 - T/T_c)$. Their relative contribution to the anisotropy of H_{c2} therefore vanishes at $T \to T_c$. The anisotropy we observe is temperature independent so cannot simply be attributed to corrections of this origin.

Irreducible representations for odd parity superconducting states for the point group D_{6h}

Table S1 summarises the different odd parity superconducting states possible for D_{6h} hexagonal symmetry. We focus on the classification of states for strong spin-orbit coupling relative to k_BT_c. The E_{1u} state is of particular interest since it describes polar states that would have a large effective mass anisotropy.

Landau expansion of the free energy for the two-dimensional Irr Reps of D_{6h}.

The uniform part of the Free energy to quartic order in $\vec{\eta} \equiv (\eta_x, \eta_y)$ contains two quartic terms with coefficients β_1 and β_2

$$F_{\text{uniform}} = \alpha(1 - T/T_c)\vec{\eta}^*.\vec{\eta} + \beta_1(\vec{\eta}^*.\vec{\eta})^2 + \beta_2\,|\vec{\eta}.\vec{\eta}|^2\,.$$

$\beta_1 + \beta_2 > 0$ is required to ensure stability; otherwise the expansion has to include higher order terms. For weak coupling the quartic terms of F_{uniform} are proportional to[17] $\int d\Omega_{\mathbf{k}} Tr(\tilde{\Delta}(\mathbf{k})^\dagger\tilde{\Delta}(\mathbf{k})\tilde{\Delta}(\mathbf{k})^\dagger\tilde{\Delta}(\mathbf{k}))$ where $\tilde{\Delta}(\mathbf{k}) = \eta_x\tilde{\Delta}_1(\mathbf{k}) + \eta_y\tilde{\Delta}_2(\mathbf{k})$ with matrices $\tilde{\Delta}_i$ related to basis vectors $\vec{d_i}$ (given in the table) by

$$\tilde{\Delta}_i = \begin{pmatrix} -(d_i)_x(\mathbf{k}) + \mathrm{i}(d_i)_y(\mathbf{k}) & (d_i)_z(\mathbf{k}) \\ (d_i)_z(\mathbf{k}) & (d_i)_x(\mathbf{k}) + \mathrm{i}(d_i)_y(\mathbf{k}) \end{pmatrix}.$$

For the 2D Irr Reps with $\vec{d_i} \parallel c$ and weak coupling $\beta_2/\beta_1 = 0.5$, whereas for $\vec{d_i} \perp c$, $\beta_2/\beta_1 = -0.5$. The ratios will differ from these values for stronger coupling and intermediate directions of $\vec{d_i}$.

The calculation of H_{c2} requires gradient terms in the Ginzburg Landau free energy. The gradient terms are the same for both 2D Irr Reps and are given by

$$F_{\text{Grad}} \;=\; K_1\,(D_i\eta_j)^*\,(D_i\eta_j) + K_2\,(D_i\eta_i)^*\,(D_j\eta_j) + K_3\,(D_i\eta_j)^*\,(D_j\eta_i) + K_c\,(D_z\eta_i)^*\,(D_z\eta_i)$$

with $i = \{x, y\}$ and $\vec{D} = \vec{\partial} + i(2\pi/\phi_0)\vec{A}$.

The K_1 term (called K below) is isotropic in the plane, $K_2 + K_3$ ($\equiv 2KC_1$) couples gradients of $\eta_+ = (\eta_x + \mathrm{i}\eta_y)/\sqrt{2}$ and $\eta_- = (\eta_x - \mathrm{i}\eta_y)/\sqrt{2}$, while $K_2 - K_3$ ($\equiv 2KC_2$) breaks the symmetry between terms with uniform η_+ and uniform η_- in a magnetic field. Stability requires $|C_2 - C_1| < 1$ and $|C_1 + C_2| < 1 + 2C_1$. Outside this range higher order derivatives have to be considered.

Solutions for H_{c2} are found from the linearised Ginzburg-Landau equations; the solution giving the maximum critical field determines H_{c2}. The maximum H_{c2} for $B \parallel c$ ($B_z > 0$) is found[18] for $(\eta_+, \eta_-) = \{L_0, 0\}$ if $C_2 > C_1^2/(1+C_1)$ with L_0 the lowest Landau level function of coordinates x, y. In the opposite limit the solution is of the form $(\eta_+, \eta_-) = \{L_2, cL_0\}$, where c depends on K, C_1, C_2 and L_2 is the second Landau level function (the mth Landau level function is $\eta_m(\rho)e^{\pm im\phi}$ expressed in polar coordinates (ρ, ϕ)

D_{6h} Irreducible Representations

Irr. Rep.	$\vec{d}_{x,y}(\mathbf{k})$ (strong SO)	$\vec{d}_z(\mathbf{k})$ (strong SO)	$\mathcal{Y}(\mathbf{k})$ (weak SO)
A_{1u}	$\begin{pmatrix} k_x \\ k_y \\ 0 \end{pmatrix}$	$\begin{pmatrix} 0 \\ 0 \\ k_z \end{pmatrix}$	N/A
A_{2u}	$\begin{pmatrix} k_y \\ -k_x \\ 0 \end{pmatrix}$	$\begin{pmatrix} 0 \\ 0 \\ k_x k_y k_z (k_x^2 - 3k_y^2)(k_y^2 - 3k_x^2) \end{pmatrix}$	k_z
B_{1u}	$\begin{pmatrix} (k_x^2 - k_y^2)k_z \\ -2k_x k_y k_z \\ 0 \end{pmatrix}$	$\begin{pmatrix} 0 \\ 0 \\ k_y(k_y^2 - 3k_x^2) \end{pmatrix}$	$k_x(k_x^2 - 3k_y^2)$
B_{2u}	$\begin{pmatrix} -2k_x k_y k_z \\ (k_y^2 - k_x^2)k_z \\ 0 \end{pmatrix}$	$\begin{pmatrix} 0 \\ 0 \\ k_x(k_x^2 - 3k_y^2) \end{pmatrix}$	$k_y(k_y^2 - 3k_x^2)$
E_{1u}	$\begin{pmatrix} k_z \\ 0 \\ 0 \end{pmatrix}; \begin{pmatrix} 0 \\ k_z \\ 0 \end{pmatrix}$	$\begin{pmatrix} 0 \\ 0 \\ k_x \end{pmatrix}; \begin{pmatrix} 0 \\ 0 \\ k_y \end{pmatrix}$	k_x; k_y
E_{2u}	$\begin{pmatrix} k_x \\ -k_y \\ 0 \end{pmatrix}; \begin{pmatrix} k_y \\ k_x \\ 0 \end{pmatrix}$	$\begin{pmatrix} 0 \\ 0 \\ (k_x^2 - k_y^2)k_z \end{pmatrix}; \begin{pmatrix} 0 \\ 0 \\ 2k_x k_y k_z \end{pmatrix}$	$k_z(k_y^2 - k_x^2)$; $k_x k_y k_z$

Table S1: The first column lists the symbols for the irreducible representations (Irr Reps) for odd parity superconductivity for the point group D_{6h}. There are 4 1D Irr Reps and 2 2D Irr Reps. The second and third columns give basis state(s) with the lowest harmonic orbital ($\mathbf{k}$) dependence for the case of strong spin-orbit coupling (compared with the average magnitude of the gap)[15]. Column two gives states with $\vec{d}$ in the basal plane and column three with $\vec{d} \parallel \mathbf{c}$. $\mathbf{k}$ is a unit direction vector in (momentum) space. A linear combination of $\vec{d} \perp \mathbf{c}$ and $\vec{d} \parallel \mathbf{c}$ basis states is also a valid basis. For the 2D Irr Reps E_{1u} and E_{2u}, the two entries separated by a semicolon have degenerate T_c. The amplitudes of the two basis states are denoted η_x and η_y and the basis states $\vec{d}_1$ and $\vec{d}_2$ in the text. Column 4 gives the lowest harmonic orbital part of the order parameter $\mathcal{Y}(\mathbf{k})$ for no spin-orbit interaction[16]; in this case the direction of $\vec{d}$ is completely unconstrained at T_c.

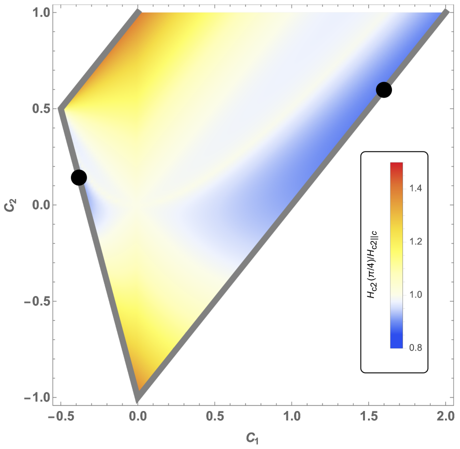

Figure S4: The figure shows the calculated H_{c2} at an intermediate angle ($\theta = \pi/4$) relative to the values at $H_{c2\|c} = H_{c2\|a}$ (fixed through the choice of K/K_c) plotted as a function of the gradient coefficients C_1 and C_2. In the blue regions $H_{c2}(\pi/4)$ is reduced relative to the value along the principle axes. The maximum reduction of $\sim 12\%$ is obtained at the points marked with large discs. Grey lines delimit the range of parameter values required for stability. Outside this range higher order gradient coefficients (quartic and above) are required.

with $\eta_m(\rho)$ the solution of $\left\{-\frac{1}{\rho}\frac{\partial}{\partial\rho}\left(\rho\frac{\partial}{\partial\rho}\right)+m^2\right\}\eta_m = \frac{2\pi B_z}{\phi_0}\eta_m$).

The value of $H_{c2} \parallel \mathbf{c}$ (close to T_C) is

$$H_{c2\|} = \frac{\phi_0}{2\pi\xi^2_{GL\perp}}(1-T/T_C)\frac{1}{(1+C_1-C_2)} \qquad \text{for } C_2 > \frac{C_1^2}{1+C_1} \quad (1)$$

$$= \frac{\phi_0}{2\pi\xi^2_{GL\perp}}(1-T/T_C)\frac{1}{3(1+C_1)-\sqrt{8C_1^2+(2+2C_1-C_2)^2}} \qquad \text{for } C_2 < \frac{C_1^2}{1+C_1}. \quad (2)$$

The Ginzburg-Landau coherence length in the plane $\xi_{GL\perp}$ is given by $\xi^2_{GL\perp} = K/\alpha$ (α is the coefficient of the homogeneous quadratic term in the Free energy). For $C_1 = 1$ and $C_2 = 0$ this gives $H_{c2\|} \sim 0.91\frac{\phi_0}{2\pi\xi^2_{GL\perp}}$.

Solutions for H_{c2} directed in the basal plane do not mix the two components η_x and η_y, resulting in states in which one component is zero. $\vec{\eta} \parallel H$ is preferred for $C_1 > 0$, and

$$H_{c2\perp} = \frac{\phi_0}{2\pi\xi_{GL\|}\xi_{GL\perp}}(1-T/T_C), \quad (3)$$

which is independent of the values of C_1 and C_2, with $\xi^2_{GL\|} = K_c/\alpha$. $\vec{\eta} \perp H$ is preferred for $C_1 < 0$ and H_{c2} is enhanced by an additional factor $1/\sqrt{1+2C_1}$.

Zhitomirsky[18] studied the cross-over between the two solutions for $H_{c2} \parallel c$ and $H_{c2} \perp c$ close to T_c as a function of the field angle. The solutions contain a mixture of all Landau levels at intermediate angles and the calculation has to be done numerically in general. Zhitomirsky solved this analytically

for $C_1 << 1$ and $C_2 = 0$. Zhitomirsky's formula is (correcting some typos):

$$f = \frac{\cos(\theta)}{\sqrt{\cos(\theta)^2 + \frac{K_c}{K(1+C_1)}\sin(\theta)^2}} \tag{4}$$

$$\Lambda_1 = 1 + C_1 - \frac{|C_1|}{2}(1 - f^2) \tag{5}$$

$$\Lambda_2 = 1 + C_1 - \frac{C_1^2(1+f)^4}{8(1+C_1)} \tag{6}$$

$$H_{c2}(\theta) = \frac{\phi_0}{2\pi\xi_{GL\perp}^2}(1 - T/T_C)\frac{1}{\sqrt{\cos(\theta)^2 + \frac{K_c}{K(1+C_1)}\sin(\theta)^2}} \times \tag{7}$$

$$\frac{1}{\frac{1}{2}\left(\Lambda_1 + \Lambda_2 - \sqrt{(\Lambda_1 - \Lambda_2)^2 + \frac{|C_1|^3(1+f)^4(1-f^2)}{8(1+C_1)}}\right)} \tag{8}$$

Note that for $\theta = 0$ we have $f = 1$, and for $\theta = \pi/2$ $f = 0$. These expressions only give the leading terms in an expansion in C_1. For $C_1 = 0$ the expression gives a simple effective mass formula, which is standard behaviour for a single component order parameter. For finite C_1 the formula predicts a reduction in H_{c2} at intermediate angles for an appropriate choice of K_c/K. For larger C_1 and $C_2 \neq 0$ a numerical solution to the eigenvalue problem is required. We have calculated numerically the relative variation of H_{c2} with angle at T_c, which depends on three parameters K_c/K, C_1 and C_2, based on the method described by Zhitomirsky (correcting what we believe is a typo in the expression he gives for $b_{n,n+2}$; in the second numerator for this coefficient in his paper, $(2n+1)(1+f)^2 \to (2n+5)(1-f)^2)$. We fixed K_c/K as a function of C_1 and C_2 to obtain $H_{c2\parallel} = H_{c2\perp}$ and adjust C_1 and C_2 over their allowed range. The resulting value of $H_{c2}(\theta = \pi/4)/H_{c2}(\theta = 0, \pi/2)$ is plotted in FIG S4, keeping terms up to the 10th Landau level (the result converges rapidly). $H_{c2}(\theta = \pi/4)/H_{c2}(\theta = 0, \pi/2) < 1$ over a wide range of parameters with an absolute minimum at the boundary imposed by the stability requirement. Beyond the boundary quartic and higher order gradients are needed for stability, which would necessitate an increase in the number of parameters. For parameters in the allowed range we find a maximum dip in $H_{c2}(\theta)$ of 12%.

For weak coupling the ratio of the gradient terms $K_{m,n,j,l}(D_m\eta_j)^*(D_n\eta_l)$ can be deduced[19] from $K_{m,n,j,l} \propto \int d\Omega_{\mathbf{k}} k_m k_n \mathrm{Tr}(\tilde{\Delta}_j^\dagger(\mathbf{k})\tilde{\Delta}_l(\mathbf{k}))$. Thus, for weak coupling $K_2 = K_3 = 0$ (ie $C_1 = C_2 = 0$) for E_{2u} for any direction of $\vec{d}$. For E_{1u}, $K_2 = K_3 = 0$ for $\vec{d} \perp \mathbf{c}$, but $K_2 = K_3 \neq 0$ if $\vec{d}$ has a component out of the plane with $K_2 = K_3 = K_1$ (ie $C_1 = 1$, $C_2 = 0$) for $\vec{d} \parallel \mathbf{c}$. $C_2 \neq 0$ is possible when there is particle hole asymmetry[20] with $C_2 \sim (T_c/E_f)^2 << 1$. For E_{1u} with $\vec{d}$ inclined (slightly) out of the plane $0 < C_1 < 1$ and $C_2 \sim 0$. These parameters give a clear dip in H_{c2} at intermediate field angles.

Two component superconductivity

Here we consider two scaler order parameters belonging to the same Irr Rep linked with a Josephson type coupling $\epsilon(\eta_1^*\eta_2 + \eta_2^*\eta_1)$. The free energy up to quadratic order in $\eta_{1,2}$ is

$$F_{\text{two band}} = \alpha_1(T)|\eta_1|^2 + \alpha_2(T)|\eta_2|^2 + \epsilon(\eta_1^*\eta_2 + \eta_1\eta_2^*) + \sum_{i=x,y,z} \frac{(m_1^{-1})_{ii}}{2}|D_i\eta_1|^2 + \frac{(m_2^{-1})_{ii}}{2}|D_i\eta_2|^2 .$$

H_{c2} for this free energy has been calculated by a number of authors in the context of two band superconductivity, including for MgB_2[21] and $NbSe_2$[22]. We consider an accidental degeneracy with $\alpha_1(T) = \alpha_2(T)$ in the following. As described in the main text, if $\epsilon = 0$ the two bands are independent and the critical field is determined by the higher of two independent critical fields which can change as a function of field angle and can result in a dip in $H_{c2}(\theta)/H_{c2}(0)$ at intermediate angles. For non zero ϵ, H_{c2} can be found numerically by expanding the solutions of the coupled Ginzburg Landau equations as a sum of uncoupled Landau-level eigenstates[21]. We find that ϵ suppresses the dip very close to T_c and results in a strong temperature dependence of the anisotropy. However, if ϵ is sufficiently small the suppression of the dip in $H_{c2}(\theta)$ may occur too close to T_c to be observable experimentally.

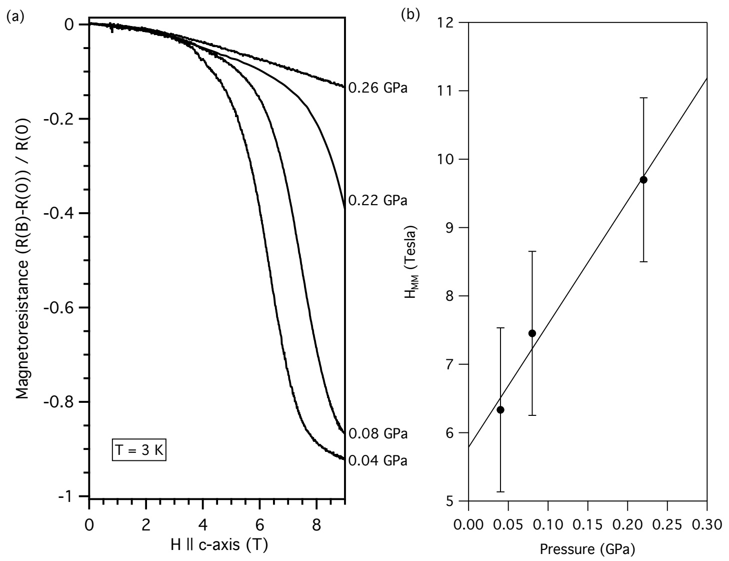

Figure S5: Panel (a) shows the magnetoresistance of a single crystal of UAu_2 at different low pressures. The drop in magneto-resistance is due to a transition to ferrimagnetism. Panel (b) shows the transition field as a function of pressure. The transition field increases strongly with pressure.

Transition to Ferrimagnetism

At ambient pressure for a field applied along the c-axis a transition from antiferromagnetism to ferrimagnetism occurs at a metamagnetic transition field $H_{MM} = 6$ T at 3 K, accompanied by a sharp drop in the electrical resistance[1]. The transition field is almost constant at low temperature but a large ($\sim \pm 2T$) hysteresis between increasing and decreasing field opens up at lower temperature. The magnetoresistance in increasing field $(\rho(H) - \rho(0))/\rho(0)$ at 3 K, which avoids this hysteresis, for a series of low pressures, is shown in FIG S5. A pressure of 0.26 GPa pushes the transition to above 9 T at 3 K. These measurements were carried out in a piston-cylinder type pressure cell adapted to applying smaller pressure than the diamond anvil cell used for the other measurements. The pressure was determined at low temperature from the resistance change of a manganin wire coil. The sample investigated was a single crystal of dimensions $1 \times 1 \times 0.5$ mm with a similar residual resistance ratio at ambient pressure to the other samples.

References

[1] O'Neill, C. D. *et al.* Non-fermi liquid behaviour below the néel temperature in the frustrated heavy fermion magnet UAu_2. *Proc. Nat. Acc. Sci.* **118**, e2102687118 (2021).

[2] Carbotte, J. P. Properties of boson-exchange superconductors. *Rev. Mod. Phys.* **62**, 1027–1157 (1990).

[3] Bulaevski, L. N., Dolgov, O. V. & Ptitsyn, M. O. Properties of strong coupled superconductors. *Phys. Rev. B* **38**, 11290–11295 (1988).

[4] Gor'kov, L. P. Superconductivity in heavy fermion systems. *Sov. SCI. Rev. A. Phys.* **9**, 1–116 (1987).

[5] Clogston, A. M. Upper limit for the critical field in hard superconductors. *Phys. Rev. Lett.* **9**, 266–267 (1962).

[6] Buzdin, A. I. & Brison, J. P. New solutions for the superconducting order parameter in a high magnetic field. *Phys. Lett. A* **218**, 359–366 (1996).

[7] Maki, K. Effect of pauli paramagnetism on magnetic properties of high-field superconductors. *Phys. Rev.* **148**, 362–369 (1966).

[8] Millis, A. J., Sachdev, S. & M.Varma, C. Inelastic scattering and pair breaking in anisotropic and isotropic superconductors. *Phys. Rev. B* **37**, 4975–4986 (1988).

[9] Schmiedeshoff, G. M., Fisk, Z. & Smith, J. L. Upper critical fields of the heavy-fermion superconductor UBe_{13}. *Phys. Rev. B* **45**, 10544–10548 (1992).

[10] Glémot, L., Brison, J. P., Flouquet, J., Buzdin, A. I. & Jaccard, D. Strong coupling superconductivity in heavy fermion systems. *Physica C* **317-318**, 73–81 (1999).

[11] Shimizu, Y., Braithwaith, D., Aoki, D., Salce, B. & Brison, J.-P. Spin-triplet p-wave superconductivity revealed under high pressure in UBe_{13}. *Phys. Rev. Lett.* **122**, 067001 (2019).

[12] Howald, L., Knebel, G., Aoki, D., Lapertot, G. & Brison, J. P. The upper critical field of $CeCoIn_5$. *New J. of Phys.* **13**, 113039 (2011).

[13] Teichler, H. On the H_{c2} in uniaxial superconductors. *Phys. stat. sol. (b)* **72**, 211–219 (1975).

[14] Mineev, V. Superconductivity in UPt_3. *Ann. Phys. Fr.* **19**, 367–384 (1994).

[15] Joynt, R. & Taillefer, L. The superconducting phases of UPt_3. *Rev. Mod. Phys.* **74**, 235–294 (2002).

[16] Tsutsumi, Y., Machida, K., Ohmi, T. & Ozaki, M. A spin triplet superconductor UPt_3. *J. Phys. Soc. Jpn.* **81**, 074717–074717(11) (2012).

[17] Mineev, V. P. & Samokhin, K. V. *Introduction to Unconventional Superconductivity* (Gordon and Breach, 1999).

[18] Zhitomirsky, M. Upper critical fields and corresponding phases in superconductors with multicomponent order parameters. *Sov. Phys. JETP* **70**, 760 (1990).

[19] Sigrsit, M. & Ueda, K. Phenomenological theory of unconventional superconductivity. *Rev. of Mod. Phys.* **63**, 239–311 (1991).

[20] Sauls, J. A. The order parameter for the superconducting phases of UPt_3. *Advances in Physics* **43**, 113–141 (1994).

[21] Dao, V. H. & Zhitomirsky, M. E. Anisotropy of the upper critical field in MgB_2: the two gap Ginzburg-Landau theory. *Eur. Phys. J. B* **44**, 183–188 (2005).

[22] Huang, H., Liu, P., Lv, P.-L. & Li, N. Theoretical study of the upper critical field of a layered superconductor $NbSe_2$. *J. Low Temp. Phys.* **177**, 217–225 (2014).
